# Reporting Quality of Oral TCM Systematic Reviews Based on the PRISMA Harms Checklist from 2013 to 2020

**DOI:** 10.1155/2023/4612036

**Published:** 2023-01-24

**Authors:** Tianying Chang, Yingzi Cui, Ying Zhang, Jinhui Ma, Jing Tan, Jian Wang

**Affiliations:** ^1^EBM Office, The Affiliated Hospital to Changchun University of Chinese Medicine, Changchun 130021, China; ^2^Neurology Department, The Affiliated Hospital to Changchun University of Chinese Medicine, Changchun 130021, China; ^3^Department of Health Research, Evidence, and Impact, McMaster University, Hamilton, ON, Canada; ^4^Chinese Evidence-Based Medicine Center and National Clinical Research Center for Geriatrics, West China Hospital, Sichuan University and Collaborative Innovation Center, Chengdu, Sichuan 610041, China

## Abstract

**Background:**

Systematic reviews focusing on the effectiveness of different kinds of healthcare interventions have been widely published, but there were few guidelines for reporting safety concerns before 2016. The PRISMA harms checklist, which was published in 2016, can standardize reporting quality.

**Objectives:**

To evaluate the safety information reporting quality of oral traditional Chinese medicine (TCM) in systematic reviews before and after the PRISMA harms checklist was published and to explore factors associated with better reporting.

**Methods:**

We searched PubMed, the Cochrane Library, and Embase to identify all systematic reviews using oral TCM as interventions published before (from 2013 to 2015) and after (from 2017 to 2020) the PRISMA harms checklist was published. We used the PRISMA harms checklist to assess the quality of reporting of the safety information to included systematic reviews.

**Results:**

In total, 200 systematic reviews were sampled from eligible studies published between 2013 and 2020. Reviews from 2016 were excluded. Scores on the PRISMA harms checklist (23 items) ranged from 0 to 12. A systematic reviews published after 2016 had better reporting quality compared with studies published before 2016 with regard to the title (*P*=0.03), results of individual studies (*P*=0.016), and risk of bias across studies (*P*=0.043). In all included systematic reviews of our study, the state conclusion in coherence with review findings was reported adequately with the proportion of adherence at 95%; other items had a reporting proportion ranging from 0% to 57%. The four essential reporting items of the PRISMA harms checklist also had a low reporting quality ranging from 0% to 4%.

**Conclusions:**

Oral TCM systematic reviews reported inadequate safety information before and after the PRISMA harms checklist was published. This survey suggested that the PRISMA harms checklist should be recommended more to both original research and systematic review authors.

## 1. Introduction

A large number of systematic reviews in the field of traditional Chinese medicine (TCM) are published every year [1]. As the original research's primary purpose was to evaluate the effectiveness of TCM, the leading outcome indicators of most systematic reviews mainly focus on effectiveness. However, randomized controlled trials can also identify common adverse reactions (drug safety events), which is often a secondary aim of these studies. Thus, many original research studies and systematic reviews do not obtain first-hand information on drug safety. Moreover, it is rare and challenging to perform meta-analysis due to the inconsistencies among side effects. This is very unfavorable to the value of existing systematic reviews, which can guide clinical practice regarding safety concerns.

Before the 2016 Preferred Reporting Items for Systematic Reviews and Meta-Analysis (PRISMA) harms checklist was published [2], most systematic review guidance was directed toward the evaluation of effectiveness. Most systematic reviews focused on the effectiveness of TCM interventions [3–10]. With concerns about drug safety issues, systematic reviews and meta-analyses of clinical trial safety data have become more important [11–14].

Traditional Chinese herbal medicine refers to the decoction of herbal medicines extracted according to TCM theory, which is based on the notion of harmony and balance [15]. It also includes herb extracts and patented herbal medicines. The drug safety information for these decoctions is essential for clinicians, especially for those TCM interventions that have a complicated effect [16, 17]. Most TCM patent medicine instructions claim the adverse reactions are not clear, and randomized controlled trials of TCM decoctions often report unknown safety issues [18, 19]. This is mainly the case when adverse drug effects are rare [20–22] when assessing drug safety [14].

Four essential reporting elements (whether or not harms were reported in the title, synthesis of results (zero events handling), study characteristics, and results synthesis) have been added to the PRISMA statement to improve harms reporting in reviews since 2016 [2]. The PRISMA-harms checklist identifies and provides a minimal set of items that should be reported when reviewing adverse events. We conducted the survey to investigate the quality of safety reporting among oral TCM systematic reviews before and after the PRISMA harms checklist were published to assess the checklist's effectiveness.

## 2. Methods

### 2.1. Eligibility Criteria

We included a study if it was a systematic review that assessed the efficacy/effectiveness of an oral TCM decoction, including granules and extracts, or an oral TCM patent. Other kinds of systematic reviews, such as network meta-analysis, individual participant data meta-analysis, and overviews of systematic reviews were excluded.

A study was defined as a systematic review according to the Cochrane Handbook criteria (version 5.1.0) [23]. Nonrandomized studies in systematic reviews included quasi-randomized clinical trials (quasi-RCTs), cohort studies, and case-control studies.

### 2.2. Information Sources and Study Selection

**Figure 1:**
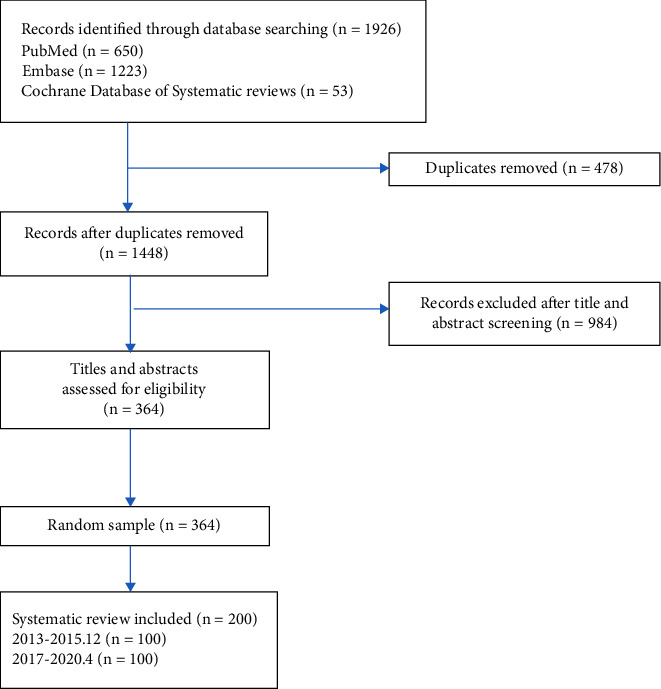
Flow chart of study selection.

Two independent authors systematically searched PubMed, Embase, and the Cochrane Library to identify systematic reviews. We searched studies from 2013 to 2015 to evaluate the quality of safety reporting before the PRISMA harms checklist was published. In addition, we also searched for studies from 2017 to April 2020 to evaluate safety reporting quality after the PRISMA harms checklist was published. The literature screening process is shown in Figure 1. The full search strategy used in Embase is shown as follows:

1. Traditional Chinese Medicine.mp. or Chinese Medicine.
2. Chung I Hsueh.mp.
3. Hsueh, Chung I.mp.
4. Chinese Medicine/or Traditional Medicine, Chinese.mp.
5. Chinese Medicine/or Zhong Yi Xue.mp.
6. Chinese Medicine/or Chinese Traditional Medicine.mp.
7. Chinese Medicine/or Chinese Medicine, Traditional.mp.
8. Chinese Medicine/or Traditional Tongue Diagnosis.mp.
9. Chinese Medicine/or Tongue Diagnoses, Traditional.mp.
10. Tongue Diagnosis, Traditional.mp. or Chinese Medicine/
11. Chinese Medicine/or Traditional Tongue Diagnoses.mp.
12. Chinese Medicine/or Traditional Tongue Assessment.mp.
13. Tongue Assessment.mp.
14. Chinese Medicine/or Traditional Tongue Assessments.mp.
15. Systematic review.mp. or “systematic review”.
16. Meta-analysis.mp. or meta-analysis.
17. Meta-analysis.mp. or meta-analysis.
18. 1 or 2 or 3 or 4 or 5 or 6 or 7 or 8 or 9 or 10 or 11 or 12 or 13 or 14,
19. 15 or 16 or 17.
20. 18 and 19.
21. Limit 20 to (English language and yr = “2017–Current”).

We conservatively estimated that 200 reports would be sufficient to obtain a robust result [14]. A total of 100 citations were sampled from studies dating from 2013 to 2015. The PRISMA harms checklist was published on 11 December 2015, so another 100 citations were chosen from studies published from 2017 to April 2020. Studies published in 2016 were excluded from the search.

### 2.3. Data Extraction and Management

Data extraction was performed by reviewer Tianying Chang. Jing Tan, a second independent reviewer, cross-checked the extraction for accuracy. We collected the following information from each included study: [1] author, [2] published year, [3] number of studies included in the review, [4] number of subjects involved in the included systematic review, [5] type of TCM intervention (decoction, extract, or patent), [6] type of control (placebo, standard of care, or other TCM intervention), and [7] type of funding.

The safety information of all included systematic reviews was evaluated by the PRISMA harms checklist [2]; we also assessed the reporting quality of all included studies with PRISMA [24]. The two independent authors assessed whether or not the PRISMA and PRISMA harms checklist items were reported.

The PRISMA harms checklist has 23 reporting items based on the PRISMA statement. Of these 23 items, four are essential, or minimum, reporting items. These four essential items include the title, synthesis of results, study characteristics, and synthesis of results. [2] The minimum item of the title (item 1) includes “specifically mention ‘harms' or other related terms, or the harm of interest in the review,” item of synthesis of results (item 14) includes “specify how zero events were handled, if relevant,” item of study characteristics (item 18) includes “define each harm addressed. How it was ascertained,” and item of synthesis of results (item 21) includes “describe any assessment of possible causality.”

All items from the PRISMA statement and the PRISMA harms checklist were evaluated and reported with “Yes” and “No.” To calculate a total score for each assessed study, all “Yes” responses were assigned a value of 1 and all “No” responses were assigned a value of 0.

### 2.4. Statistical Analysis

We conducted a statistical description for the reporting items of all included systematic reviews. Dichotomous variables were described with frequencies and percentages. Continuous variables were described with means and medians. We compared the characteristics of systematic reviews before and after the PRISMA harms checklist was published. Dichotomous variables were tested with a chi-square test, and continuous variables were tested by the *t*-test when the distribution was normal.

## 3. Results

The initial systematic search resulted in a total of 1926 citations from PubMed, Embase, and the Cochrane Library. After 478 duplicate publications were removed, the titles and abstracts of 1448 records were screened, and 984 irrelevant records were excluded. After assessing the full texts, 364 systematic reviews satisfied the eligibility criteria and were included in the critical evaluation (Figure 1). We sampled 200 studies from 364 included reviews. Twenty-one Cochrane systematic reviews were chosen before randomization as having better review quality. In total, 100 systematic reviews were published from January 2013 to December 2015, and another 100 systematic reviews were published from January 2017 to April 2020.

The median number of studies included among the eligible systematic reviews was 12 (ranging from 0 to 83). The median number of participants included in the studies was 1081 (ranging from 0 to 8138). All studies assessed the effects of oral TCM preparations [14]. The range of PRISMA scores was 18–27 in publications before 2016, and 11–27 in publications after 2016. The average scores were 23.4 in publications before 2016 and 22.9 in publications after 2016.

### 3.1. Characteristics of Included Studies

Among the 100 systematic reviews published before 2016, four studies included RCTs and quasi-RCT [25–28]; the remainder of the studies included RCTs. In the 100 systematic reviews published after 2016, one study included RCT and a quasi-RCT [29]. Another study included a controlled trial [30], and the remaining studies included RCTs. The publication distribution of the selected studies was as follows: 38 studies (19%) were from 2013, 21 studies (10.5%) were from 2014, 41 studies (20.5%) were from 2015, 26 studies (13%) were from 2017, 31 studies (15.5%) were from 2018, 36 studies (18%) were from 2019, and 7 studies (3.5%) were from 2020.

There were 22 categories of diseases among the included studies. The top 10 were nervous system diseases (12.5%), digestive system diseases (11.5%), oncological diseases (9%), cardiac diseases (8%), hypertension (7%), dermatological disease (6.5%), endocrine and metabolic diseases (6.5%), urinary system disease (6%), immune system diseases (5.5%), and respiratory diseases (5.5%).

The first authors of all included studies were from 84 hospitals/universities. Methodologists were identified by the co-authors' backgrounds in epidemiology, biostatistics, and evidence-based medicine. Among the 100 systematic reviews published before 2016, methodologists participated in 66 studies (66%). Among the 100 systematic reviews published after 2016, methodologists participated in 82 (82%) studies. Of the included studies, a total of 178 studies were from mainland China, 8 were from Australia, 6 were from Hong Kong, 1 was from Macau, 2 were from Korea, 1 was from Malaysia, 2 were from Singapore, 1 was from the United Kingdom, and 1 was from the Netherlands.

### 3.2. Adherence to the PRISMA Harms Checklist

**Figure 2:**
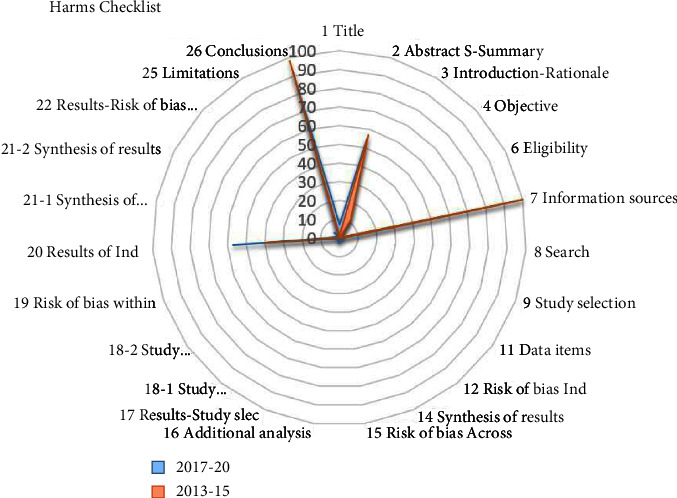
Adherence to the PRISMA harms checklist before and after its publication (%).

Most systematic reviews met a few requirements of the PRISMA harms checklist (Table 1 and Figure 2). Among the 23 items on the PRISMA harms checklist, only one item (conclusions‐statement of conclusions in coherence with the review findings) was reported adequately (proportion of adherence = 95%). The proportion of reporting other criteria ranged from 0% to 57%. For the four essential reporting items, 8 (4%) reviews specifically mentioned “harms,” other related terms or the harm of interest in the review title. A total of three reviews (1.5%) specified how zero events were handled. Two reviews (1%) defined each harm that how it was ascertained, and over what time period; 0 reviews (0%) described the assessment of possible causality.

In the analysis by the Pearson's chi-squared test, systematic reviews published after 2016 had a better reporting quality with regard to title s(7% vs. 1%, *P*=0.03), results of individual studies (40% vs. 57%, *P*=0.016), and risk of bias across studies (0% vs. 4%, *P*=0.043). In other items, there was no statistical difference.

## 4. Discussion

In this survey, we found that the reporting quality of safety information among oral TCM systematic reviews was generally low before and after the PRISMA harms checklist was published. We did not include studies from 2016 because the PRSIMA harms checklist was published in December 2015. The four essential PRISMA harm items (proportion and adherence ranging from 0% to 4%) also had a low reporting quality. Our findings for 19 nonessential items showed a proportion of adherence ranging from 0% to 95%, which is consistent with surveys of systematic reviews assessing harms reporting for various health care interventions (adherence ranging from 1.7% to 81.6% and from 13% to 62% [14, 31]). The inclusion of safety information is not the primary aim of most oral TCM systematic reviews, and, thus, the safety reporting deficiency can be somewhat attributed to that fact. Twenty-one Cochrane systematic reviews were included, but with the unbalanced number of non-Cochrane systematic reviews, we did not compare the reporting quality between each other.

Item 7 of the PRISMA harms checklist reads, “Report if you only searched for published data or also sought data from unpublished sources, from authors, drug manufacturers, and regulatory agencies. If includes unpublished data, provide the source and the process of obtaining it.” Most systematic review authors attempted to acquire all collectable data, but unpublished data were usually unavailable. If an author searched several unpublished databases but obtained no results, we question whether or not item 7 should be considered as included. Should this item be evaluated by search results or search process? The differences in how item 7 may have been considered by different reviewers are a possible question to address in a future examination.

There are several strengths in our survey. First, we systematically surveyed the reporting quality of safety information among reviews of oral TCM, which were obtained from PubMed, Embase, and the Cochrane Library. Second, the included systematic reviews of our survey were chosen over a relatively broad time span (from 2013 to 2020) and sampled, which represents a more robust survey outcome.

Limitations also exist in our survey. First, the interventions were limited to oral TCM, including decoctions (herbs, granules, and extracts) and patent medications. Other routes of TCM administration were excluded, but external TCM and TCM injections also have some adverse effects reported. TCM injections, especially some injections for the purpose of treating cancer, have reported adverse effects [32], although some adverse effects were obviously related to the cancer itself. Second, a reporting guideline requires a considerable period of time to determine if it has been implemented into practice. The implementation of the PRISMA harms checklist was hard to assess in the TCM field because of the relatively short time since implementation. Third, as network meta-analysis, overviews of systematic reviews, and individual participant meta-analysis were excluded, the findings of our survey did not generalize more information to conduct a more comprehensive outcome from these reviews. As we aimed to acquire a better study quality, we only searched for studies published in English, which have been considered to be of better quality.

The consideration of adverse effects is an essential issue in drug trials and meta-analyses [14]. The authors of systematic reviews often focus on the efficacy of interventions but do not consider safety. In TCM clinical studies and meta-analyses, adverse effects are not adequately reported [16], which could have a negative effect on medication and treatment guidance. Inadequate reporting and assessment of safety would also negatively impact guidance for clinical practice.

The reporting of safety information should be guided by well-designed analyses. The PRISMA harms checklist should be more widely promoted to systematic review authors.

## 5. Conclusions

Systematic reviews of oral TCM published before and after 2016 did not show a significant statistical difference in safety reporting quality. Both time periods demonstrated a poor usage of the PRISMA harms checklist. Our survey suggests a strong need to use the PRISMA harms checklist for reporting safety information among oral TCM systematic reviews. Systematic review authors should pay more attention to safety information reporting.

## Figures and Tables

**Table 1:** Adherence of systematic review reporting of the PRISMA harms checklist.

Sections	Items	PRISMA harms checklist			
		Total (%)	Published before 2016 (%)	Published after 2016 (%)	*P* values
Title	1 Title	8 (4)	1 (1)	7 (7)	0.03
Abstract	2 Structured summary	112 (56)	57 (57)	55 (55)	0.78
Instruction	3 Rationale	22 (11)	11 (11)	11 (11)	1.0
Methods	4 Objectives	1 (0.5)	0	1 (1)	0.32
	6 Eligibility criteria	2 (1)	1 (1)	1 (1)	1.0
	8 Search	3 (1.5)	1 (1)	2(2)	0.56
	9 Study selection	0	0	0	—
	11 Data items	2 (1)	0	2 (2)	0.155
	12 Risk of bias in individual studies	2 (1)	0	2 (2)	0.155
	14 Synthesis of results	2 (1)	0	2 (2)	0.155
	15 Risk of bias across studies	2 (1)	0	2 (2)	0.155
	16 Additional analyses	3 (1.5)	0	3 (3)	0.081

Results	17 Study selection	0	0	0	—
	18 Study characteristics-1	1 (0.5)	0	1 (1)	0.316
	18 Study characteristics-2	0	0	0	—
	19 Risk of bias within studies	2 (1)	0	2 (2)	0.155
	20 Results of individual studies	97 (48.5)	40 (40)	57 (57)	0.016
	21 Synthesis of results-1	2 (1)	0	2 (2)	0.155
	21 Synthesis of results-2	0	0	0	—
	22 Risk of bias across studies	4 (2)	0	4 (4)	0.043

Discussion	25 Limitations	1 (0.5)	0	1 (1)	0.316
	26 Conclusions	190 (95)	98 (98)	92 (92)	0.052

## Data Availability

The data used to support the findings are available from the corresponding authors upon reasonable request.
